# A Strong Immune Response in Young Adult Honeybees Masks Their Increased Susceptibility to Infection Compared to Older Bees

**DOI:** 10.1371/journal.ppat.1003083

**Published:** 2012-12-27

**Authors:** James C. Bull, Eugene V. Ryabov, Gill Prince, Andrew Mead, Cunjin Zhang, Laura A. Baxter, Judith K. Pell, Juliet L. Osborne, Dave Chandler

**Affiliations:** 1 School of Life Sciences, University of Warwick, Coventry, United Kingdom; 2 School of Life Sciences and Warwick Crop Centre, University of Warwick, Wellesbourne Campus, Wellesbourne, Warwickshire, United Kingdom; 3 Warwick Systems Biology Centre, University of Warwick, Coventry, United Kingdom; 4 Centre for Soils and Ecosystem Function, Department of Plant and Invertebrate Ecology, Rothamsted Research, Harpenden, Hertfordshire, United Kingdom; Stanford University, United States of America

## Abstract

Honeybees, *Apis mellifera*, show age-related division of labor in which young adults perform maintenance (“housekeeping”) tasks inside the colony before switching to outside foraging at approximately 23 days old. Disease resistance is an important feature of honeybee biology, but little is known about the interaction of pathogens and age-related division of labor. We tested a hypothesis that older forager bees and younger “house” bees differ in susceptibility to infection. We coupled an infection bioassay with a functional analysis of gene expression in individual bees using a whole genome microarray. Forager bees treated with the entomopathogenic fungus *Metarhizium anisopliae* s.l. survived for significantly longer than house bees. This was concomitant with substantial differences in gene expression including genes associated with immune function. In house bees, infection was associated with differential expression of 35 candidate immune genes contrasted with differential expression of only two candidate immune genes in forager bees. For control bees (i.e. not treated with *M. anisopliae*) the development from the house to the forager stage was associated with differential expression of 49 candidate immune genes, including up-regulation of the antimicrobial peptide gene *abaecin*, plus major components of the Toll pathway, serine proteases, and serpins. We infer that reduced pathogen susceptibility in forager bees was associated with age-related activation of specific immune system pathways. Our findings contrast with the view that the immunocompetence in social insects declines with the onset of foraging as a result of a trade-off in the allocation of resources for foraging. The up-regulation of immune-related genes in young adult bees in response to *M. anisopliae* infection was an indicator of disease susceptibility; this also challenges previous research in social insects, in which an elevated immune status has been used as a marker of increased disease resistance and fitness without considering the effects of age-related development.

## Author Summary

Honeybees have a highly developed form of social biology in which tasks are distributed among workers according to their age, with younger bees performing housekeeping tasks (“house bees”) before switching to foraging duties when they grow older. This division of labor is vital to colony function and survival. Pathogens are known to be partly responsible for the current decline in honeybee populations around the world, but we understand little about the responses of different types of worker bee to infection. In this study, we infected house and forager bees with an insect pathogen. We measured bee survival rate and the expression of genes that regulate the immune system. More immune genes were up regulated in house bees than foragers in response to infection, but foragers were more resistant to the pathogen than house bees. We found that development from the house to forager stages resulted in increased expression of genes that regulate the production of antimicrobial proteins. The inference is that parts of the immune system are activated during development, resulting in greater resistance to infectious disease in forager bees. Our study provides new insights into the functioning of the honeybee immune system and its interaction with social organisation.

## Introduction

Declining populations of honeybees, *Apis mellifera*, have been recorded in many countries, causing widespread concern [1], [2]. While no single factor has been found to account for all honeybee colony losses in all areas, pathogens ( = parasites that cause disease) are known to play an important role [3], [4]. Therefore, detailed understanding of the effects of pathogens on honeybee biology is critical to the development of new ways for improving bee health.

Like other eusocial insects, honeybees have a highly developed form of social organization, characterized by the presence of overlapping generations within the colony, cooperative care of offspring, and reproductive division of labor [5], [6]. Their success can be attributed to living in large, organized colonies which improves their ability to compete for resources against small groups or solitary species [7]. However, the close physical contact within the colonies of eusocial insects enables pathogens to spread rapidly [6], [8]. As a result, honeybees – like other eusocial insects – invest heavily in pathogen defense [9]. Empirical evidence indicates that selection by pathogens has been a defining feature of the evolution of insect societies [10]. The defenses used by eusocial insects against pathogens include inducible cellular and humoral immunity, antimicrobial defense compounds secreted on the cuticle, as well as group defenses that include hygienic behavior and utilization of antimicrobial compounds acquired from the environment [9], [11]–[14]. In addition, the genetic diversity within honeybee colonies is increased by polyandry (mating of the queen with multiple males), which is important to help the colony resist disease [15]–[17].

Within eusocial insect societies, functionally sterile adult workers perform most of the tasks of the colony [18]. Some tasks, such as foraging, are done later in life. These tasks are associated with greater risks, and performing them later in life has been shown to increase the average life span of individuals in the colony [19], [20]. In honeybees, adult workers born in the spring and summer spend the first part of their life inside the colony engaged in housekeeping duties such as food processing and care of brood (for this reason they are referred to as “house” bees [21]–[23]) before making a transition to foraging duties outside the colony at an average of 23 days old [24]. Foraging bees senesce rapidly and have a high mortality rate from predation [25]. The average life span of a forager bee is only five days [24]. The exact timing of the onset of foraging is affected by bee genotype [26] and also by the needs of the colony, with house bees switching to foraging duties early if the colony suffers a shortfall in forager numbers [27]. The situation is markedly different for worker bees produced in the late summer and autumn, which remain inside the colony to ensure its survival over the winter and live for approximately six months [24].

An important challenge in the study of eusociality is to understand the relationships between an individual's behavioral role, its age, its ability to withstand infection and the impact on the whole colony. Different hypotheses have been proposed about how honeybee immunity interacts with age-related division of labor. The first hypothesis states that the immunocompetence of adult bees declines markedly when they switch from housekeeping to foraging, driven by natural selection at the colony level, resulting in allocation of resources for foraging rather than immunity, both of which are energetically expensive [28]. This is supported by experiments in which a decrease was observed in the number of functional hemocytes in 26 day old forager bees compared to bees of the same age manipulated to keep them at the housekeeping behavioral stage, alongside an increase in juvenile hormone titer and a decrease in vitellogenin titer [28]. These changes were reversed if foragers were manipulated to revert to housekeeping [28]. Further support for this hypothesis comes from an observation that newly emerged house bees exhibited hemocyte nodulation reactions against bacterial challenge, whereas older, forager bees did not have this ability [29]. Finally, forager bees have a smaller fat body than one day old house bees, which may indicate a reduced ability to produce antimicrobial peptides, as the fat body is the main site of synthesis of these compounds [30].

However, there is also evidence to support a contrasting hypothesis that immunocompetence is enhanced in foragers. Natural selection may act to preserve immunity in foragers, since they are exposed to pathogens at foraging hotspots [31] and thus are a route for bringing new infections into the colony [32]. This is supported by data which showed that: (i) foragers had a significantly higher total hemocyte count than one day old house bees; (ii) there was no significant difference in the cellular encapsulation response of foragers and one day old house bees; (iii) foragers showed significantly greater phenoloxidase activity (responsible for the melanization of invading pathogen cells) than one day old house bees [30]. A refinement of this hypothesis, proposed by [33], states that cellular immunity declines in adult bees as they age, but that other parts of the immune system are maintained. This is based on experimental evidence showing that while the total hemocyte count fell in adult honeybees from one to 24 days old, phenoloxidase activity (which is involved in the melanisation and encapsulation of invading pathogens in the haemocoel) increased early in adult life and reached a plateau by the end of the first week [33]. The same patterns were observed in older foragers versus artificially produced younger foragers, and artificially produced older house bees versus younger house bees [33].

**Figure 1:**
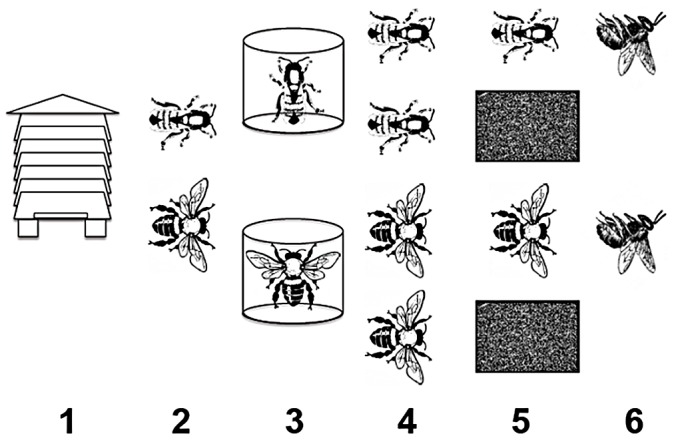
Schematic outline of experimental procedure. (1) Adult honeybees originated from a single hive with a naturally mated queen. (2) Separate cohorts of ‘house’ and ‘forager’ bees were collected and checked for signs of infection by naturally occurring pathogens. (3) Groups of bees from each cohort were infected with *Metarhizium anisopliae*, or mock infected. (4) Groups of infected and control bees were split into those destined for bioassay or microarray. (5) Bioassay bees were censused twice daily; at 48 hrs p.i., bees destined for microarray analysis were sacrificed. (6) Bioassays were maintained until all infected bees died, at which point control bioassays were censored.

Until now, controlled pathogen infection experiments linked to honeybee adult age have not been reported. Moreover, previous research has used a limited number of markers for bee immune response. For this study, we used a laboratory bioassay to quantify the susceptibility of house *vs.* forager bees from the same cohort to infection with the entomopathogenic fungus *Metarhizium anisopliae* s.l. At the same time, we quantified changes in global gene expression in individual bees using an oligonucleotide microarray constructed from the official honeybee gene set (see Figure 1 for a schematic outline of the study). We used a balanced statistical design in the microarray experiment with emphasis on maximizing the number of biological replicates per treatment, in order to determine statistically significant changes in gene expression within the experimental population. We used eight biological replicates for each of four treatments hybridized to microarrays. Findings supported our central hypothesis that selection by pathogens would result in foragers being less susceptible to infection than house bees. We went on to quantify our second hypothesis; that this difference is reflected by interpretable differences in gene expression, particularly for immune pathways. This type of combined approach tests whether strong immune responses at the molecular level are a good indicator of resistance to pathogens, and consequently fitness at the level of the whole organism.

## Results

### Pathogen bioassay: Young house bees showed greater susceptibility to infection than older forager bees

**Figure 2:**
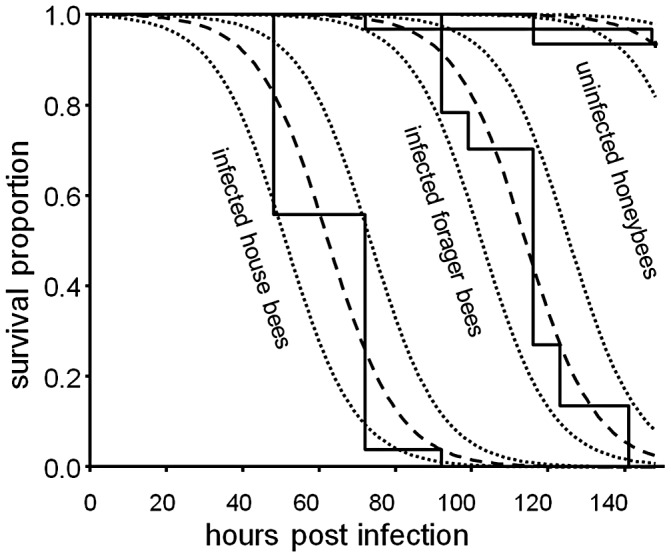
Survival analysis of worker honeybee fungal infection bioassay. Survival of honeybees following infection by *Metarhizium anisopliae* s.l. Solid lines show observed mortalities. Lines ending with “+” indicate censored populations. Dashed lines indicate expected decline in populations (dotted lines mark 95% confidence envelopes), estimated by fitting a logistic model of survival.

House bees (one day old) and forager bees (26 days old) showed differences in the rate at which they succumbed to lethal infections of the entomopathogenic fungus *M. anisopliae* s.l. in a laboratory bioassay. The median (interquartile range) observed survival time was 72 (24) hrs for *M. anisopliae*-treated house bees and 116 (24) hrs for *M. anisopliae*-treated forager bees. The *M. anisopliae*-treated forager bees survived significantly longer than *M. anisopliae*-treated house bees (*t*_149_ = 15.0, *p*<0.001) (Figure 2). Random differences within groups of biological replicates did not account for a significant amount of observed deviance (Δdeviance = 1.80, *p* = 0.097). Quantification of *M. anisopliae* 18S rRNA by RT-PCR (see supplementary information Figure S1) indicated that the fungus was present at significantly higher levels in *M. anisopliae*-treated house bees at 48 hrs post inoculation compared to *M. anisopliae*-treated forager bees (ΔC_t_ = 2.65, *t*_22_ = 14.5, *p*<0.001). The fungus was not detected in control ( = un-inoculated) bees (Figure S1).

### Transcriptomic differences were evident between pathogen-treated house and forager bees

**Figure 3:**
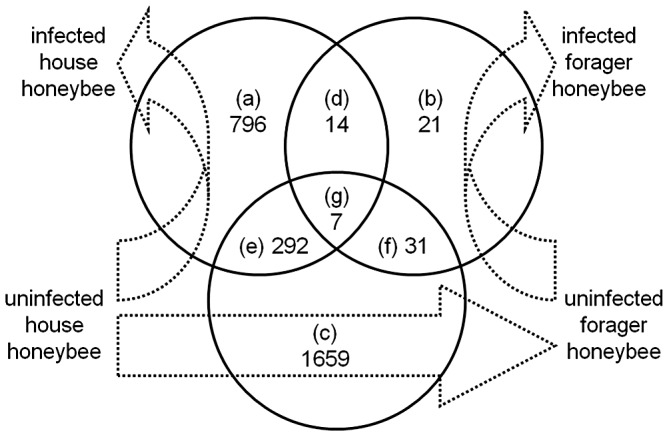
Genome wide differential expression associated with honeybee worker type and age. Venn diagrams of differential probe expression; identified at a significance probability threshold of *p*<(1/number of probes). Circles represent: a) infected with *M. anisopliae*, compared to uninfected house honeybees; b) infected, compared to uninfected forager honeybees; and c) uninfected house, compared to uninfected forager honeybees.

Genome-wide honeybee transcript abundance was quantified using microarrays 48 hrs after bees were treated with *M. anisopliae* s.l. The transcriptome data was analysed in a mixed effects model, which encompassed experimental sources of variation as structured variance components, and the presence of naturally occurring, asymptomatic honeybee viruses in individual bees as an additional covariate. We observed a significant effect of the virus covariate (deformed wing virus and/or *Varroa destructor* virus-1 or their hybrids [34]) on global gene expression of forager bees (it was not possible to deduce the effect on house bees because none of the control house bees showed high virus levels), where differences in the amount of virus detected with the microarray could account for up to a half of the variation in expression of immunity-related genes in individual forager bees following fungal infection. By comparison of statistical models including or excluding ‘virus level’, we found that virus level was associated with the differential expression of three honeybee immunity-related genes that were significantly differentially expressed as a result of *M. anisopliae* infection: *Toll-7* (GB15177), *Tube* (GB15684) and *Tep-B* (*thioester containing protein B*; GB11563). We then quantified three treatment contrasts relating to transitions between phenotypic states (younger house bee→older forager bee) and *M. anisopliae* disease states (uninfected→infected), summarized as a Venn diagram (Figure 3). There were marked differences in gene expression depending on treatment. We found that 1109 probes (representing genes) showed significant (*p*<1/*n*, where *n* = 10498 is the number of probes on the array) differential expression associated with fungal treatment of house bees (Venn diagram intersections a, d, g, e; Figure 3), while only 73 probes showed significant differential expression associated with fungal treatment of forager bees (Venn diagram intersections b, d, g, f; Figure 3). In addition, 1989 probes were differentially expressed in forager bees compared to house bees, independent of infection status (Venn diagram intersections c, e, f, g; Figure 3). Of these, there were 1659 differentially expressed genes that were uniquely associated with ageing in untreated bees (Venn diagram intersection c; Figure 3).

In order to test hypotheses on the role of age-related division of labor in honeybees in response to infection with *M. anisopliae*, we went on to identify differentially expressed probes associated with *M. anisopliae* treatment which were either unique or common to house and forager bees. House bees treated with *M. anisopliae* showed 1088 (589 up-regulated, 499 down-regulated, Table 1) differentially expressed probes that were not differentially expressed in *M. anisopliae*-treated forager bees (Venn diagram intersections a, e; Figure 3). In contrast, there were 52 (29 up-regulated, 23 down-regulated, Table 1) differentially expressed probes in forager bees that were not found to change in house bees (Venn diagram intersections b, f; Figure 3). Only 21 probes showed differential expression in response to *M. anisopliae* treatment that were common to house and forager honeybees (Venn diagram intersections d, g; Figure 3). Of these, the majority changed expression in the same direction (either up- or down-regulated) in both classes of worker bee (Table 1).

**Table 1:** Contingency tables of numbers of up- and down-regulated differentially expressed genes for house and forager honeybees treated with the entomopathogen *M. anisopliae* s.l.

**Venn diagram intersection d**		**uninfected→infected forager bees**		
		up-regulated	down-regulated	total
**uninfected→infected house bees**	up-regulated	11	0	11
	down-regulated	0	3	3
	total	11	3	14
**Venn diagram intersection e**		**uninfected house→uninfected forager bees**		
		up-regulated	down-regulated	total
**uninfected→infected house bees**	up-regulated	136	11	147
	down-regulated	27	118	145
	total	163	129	292
**Venn diagram intersection f**		**uninfected house→uninfected forager bees**		
		up	down	total
**uninfected→infected forager bees**	up	0	14	14
	down	17	0	17
	total	17	14	31
**Venn diagram intersection g**		**uninfected house→uninfected forager bees, up-regulated**		
		uninfected→infected forager bees		
		up-regulated	down-regulated	total
**uninfected→infected house bees**	up-regulated	1	1	2
	down-regulated	0	1	1
	total	1	2	3
		**uninfected house→uninfected forager bees, down-regulated**		
		uninfected→infected forager bees		
		up-regulated	down-regulated	total
**uninfected→infected forager bees**	up-regulated	1	0	1
	down-regulated	3	0	3
	total	4	0	4

We used qRT-PCR to quantify the level of mRNAs for honeybee *beta actin* and *vitellogenin* genes. Changes in expression for these genes were in the same direction for the microarray and the qRT-PCR. Levels of *vitellogenin* mRNA were higher in 26 day old forager bees compared to the one day old house bees (Figure S1), in accordance with previous studies [35], [36]. Levels of *beta actin* mRNA were lower in the forager bees compared to house bees (Figure S1). Treatment with *M. anisopliae* had no significant effect on levels of *vitellogenin* mRNA or *beta actin* mRNA in house or forager bees.

### Assigning biological functions to differentially expressed genes

Gene Ontology was used to examine potential biological functions of differentially expressed genes. Information was obtained through comparison with *Drosophila melanogaster* genome annotation for 6325 out of 10498 bee genes (62%). To examine functional differences related to the tested phenotypic transition states, we examined sets of genes for over-representation in biological process, molecular function and cellular component GO categories (Table S1). For the set of genes that were differentially expressed in *M. anisopliae*-treated house bees but not in forager bees (Venn diagram intersections a+e), there was over-representation (*p*<1E-6) of GO terms associated with cellular and subcellular organization and regulation. There were no significantly over-represented GO terms associated with responses to fungus that were either unique to forager bees (Venn diagram intersections b+f; Figure 3) or that were common to house and forager bees (Venn diagram intersections d+g; Figure 3). There were also differences between house and forager bees, independent of *M. anisopliae* infection (Venn diagram intersections c, e, f, g; Figure 3) associated with ageing, specifically in energy generation and DNA remodelling.

We also compared the observed differentially expressed genes in our experiment to a set of 182 previously published homology assignments made for honeybee immune-related genes [37]. A subset of these candidate immune genes showed differential expression in response to infection by *M. anisopliae* in our experiment, but there was no commonality in the pattern of response between house and forager bees (Table 2, Table S2). House bees treated with *M. anisopliae* showed 35 differentially expressed genes that were associated with immune function (Venn diagram intersections a, d, g, e; Figure 3). Of these, 20 genes were up-regulated, and 15 down-regulated. In contrast, *M. anisopliae*-treated forager bees showed only two differentially expressed genes that were associated with immune function (Venn diagram intersections b, d, g, f; Figure 3) (one up-regulated, one down-regulated). One of these two genes (*C-type lectin*; GB14265) was also differentially expressed in *M. anisopliae*-treated house bees. However, it was up-regulated in *M. anisopliae*-treated house bees whereas it was down-regulated in *M. anisopliae*-treated forager bees.

**Table 2:** Transcriptomic differences between house and forager bees: Summary of candidate immune genes (based on previous homology assignments [34]) differentially expressed in response to infection by *M. anisopliae* s.l.

	Number of differentially expressed candidate immune genes		
	uninfected infected house bees	uninfected infected forager bees	uninfected house forager bees
up-regulated	20	1	32
down-regulated	15	1	17
total	35	2	49

In controls, i.e. bees not treated with *M. anisopliae*, 49 candidate immune genes showed differential expression associated with honeybee ageing (i.e. house vs. forager bees; Venn diagram intersections c, e, f, g; Figure 3) (34 up-regulated, 16 down-regulated). Of these, 34 genes were uniquely associated with ageing (Venn diagram intersection c; Figure 3), i.e. they were not expressed in response to *M. anisopliae* infection. Of these, 20 were up-regulated and 14 were down regulated. Thirteen differentially expressed candidate immune genes were common to bee ageing and *M. anisopliae* infection of house bees (Venn diagram intersection e; Figure 3).

## Discussion

### A strong immune response in house bees as an indicator of increased susceptibility to infection

There is an urgent requirement for new knowledge on the molecular mechanisms by which honeybees interact with pathogens in order to better understand honeybee colony losses and to develop new interventions. However, conducting molecular studies with honeybees is not straightforward. Honeybee colonies are semi-wild, outdoor entities and present a number of significant challenges for experimenters. As a result of multiple matings by the queen, the worker bees within a colony are not genetically uniform [17] while background, asymptomatic virus infections are common [38]. In order to understand bee-pathogen interactions in the colony, we need experimental systems that are able to encapsulate the complexity of the bee immune response at the molecular level, ascertain the relationship between immune response and susceptibility to infectious disease, and take into account natural variation between individual bees. Studying whole genome transcriptional responses to infection provides a wider view of the honeybee-induced immune response, for example by enabling different genetic pathways to be studied in parallel. Within the limitations of the financial resources available to us for the microarray study, we designed the experiment to maximize the number of biological replicates using individual bees, as opposed to “pooling” bees into a sample. This enabled us to take into account the level of background asymptomatic virus infection in individual bees as a factor in the data analysis.

Measurements of animal immune status are often used as a “short cut” for measuring resistance to infection, based on an assumption that individuals with greater antibody levels, blood cell encapsulation response etc. are fitter and less susceptible to a pathogen [39]. Often, a small number of markers of immune status are employed. This approach has been used widely in studies of honeybee immunity [28]–[30], [33], [40], [41]. In our study, one day old house bees were more susceptible to *M. anisopliae* infection than 26 day old forager bees (i.e. they died faster and supported more growth of invading fungus) but exhibited a greater immune response. Hence, in this case, a strong induced immune response was an indicator of higher susceptibility to a pathogen rather than resistance. In contrast, a lower induced immune response in forager bees was associated with a reduced susceptibility to *M. anisopliae*, linked to bee ageing (see below). These findings suggest that the underlying assumption behind some previous honeybee studies may be wrong, i.e. the size of the induced immune response is not necessarily related to the ability to withstand infection or with host fitness [39], [42]. It is also clear from these results that the immunocompetence of foragers bees did not decline compared to house bees, as has been proposed previously [28]. The caveat is that *M. anisopliae* is a generalist entomopathogen that, although lethal to honeybees and other social insects and provides a very tractable experimental system, does not cause natural honeybee colony-scale outbreaks. There is a requirement to investigate how the immune response of house bees and foragers responds to co-evolved honeybee pathogens, such as *Nosema apis* and *Nosema cerana* (fungal pathogens that infect the midgut epithelium of adult bees and which cause epizootics within colonies) to compare against the response of *M. anisopliae* as a baseline, and to determine whether the resource allocation to immune defenses is the same for different types of pathogen. Many of the co-evolved entomopathogens of honeybees, such as the fungus *Ascosphaera apis* and the bacteria *Paenibacillus larvae* and *Melissicoccus pluton*, cause lethal infections only in brood, but their effects on adult bees are unclear [6].

### Exposure to pathogen infection was reflected by differential expression of candidate immune genes in house bees but not in forager bees

The Gene Ontology analysis provided some useful general information but did not provide the fine level of detail that we needed for new insights on honeybee immune function. This is likely to result from the lack of a genome annotation for *A. mellifera* and we suggest that this is an important objective for future work. In house bees treated with *M. anisopliae*, differentially expressed genes were over-represented by GO terms associated with cellular and organelle organization and biochemical regulation. This may reflect the effects of pathogenesis, as entomopathogenic fungi utilize a range of tactics to evade host immune response based on interference with regulatory networks, including suppression of cytoskeleton formation and other features of the subcellular structure of host immune cells [43], [44]. When forager bees were compared against house bees in the absence of *M. anisopliae* infection, there was over-representation of terms that highlighted the effects of bee ageing. The transition from house to forager bee is under hormonal control [45] and is accompanied by changes in biochemistry, physiology, neurobiology and metabolism that involve multiple pathways [46]–[48]. In our experiment, over-representation of GO terms associated with the ageing occurred in two areas: firstly in energy generation, with terms such as generation of precursor metabolites and energy, respiratory electron transport chain, and ATP synthesis coupled electron transport being significantly over-represented. Secondly, over-representation of terms such as chromatin assembly or disassembly, and nucleosome suggested DNA re-modelling during the ageing process, with a concomitant impact on DNA transcription, repair, and replication [49].

We went on to look at individual honeybee genes that have been hypothesized to function in bee innate immunity. The honeybee innate immune system is comprised of cellular defenses from specialized blood cells (granulocytes and plasmatocytes) within the haemocoel [50], [51] as well as humoral immunity in the form of Toll, Imd (immune deficiency) and Janus kinase/signal transduction and activator of transcription (JAK/STAT) pathways for the production of antimicrobial peptides, melanization of invading pathogen cells, and apoptosis [37]. Interpretation of the immune gene expression data in this study has to be done with a certain amount of caution. The current state of knowledge of individual honeybee immune pathways, and the mechanisms by which the different pathways interact, is not fully developed. We can draw on the literature on transcriptomics of the immune response from other insects, particularly *Drosophila*, but even here very few studies have been done using entomopathogens and natural routes of infection [52]–[54].

House and forager bees were at different physiological stages of *M. anisopliae* infection at the time of sampling in the bioassay, as shown by significant differences in the amount of fungal biomass detected within infected bees. This raises the question of whether the difference in immune gene expression in forager versus house bees was the cause or the consequence of reduced susceptibility to *M. anisopliae* in forager bees. Nevertheless, patterns were evident in our data that give insights into the bee innate immune system, including the identification of putative functionally-related components of the immune response. House bees showed significant differential expression of 35 candidate immune genes in response to fungal infection. Fungal infection activated both Imd and Toll signalling pathways in house bees, the major regulators of immune responses in insects [55]. Three out of five honeybee antimicrobial peptide (AMP) genes were significantly up-regulated in house bees (*abaecin*, GB18323; *Defensin-2*, GB10036; *Hymenoptaecin*, GB17538) and showed between 16 and 64 fold increases in expression, which was the highest fold change in expression of all differentially expressed immune genes. Changes in expression levels were observed for several components of the Toll pathway in house bees. The Toll pathway is associated with the immune response to fungi and bacteria in *Drosophila* and regulates the expression of AMP genes [56], [57]. Toll pathway genes up-regulated in house bees in our study included those encoding for extracellular components associated with fungal recognition (*PSH-like/cSP14*, GB14044; *NEC-like*, GB16472) and intracellular components including the NF-κB-like transcription factor *Dorsal* (GB19066). Only one out of eight genes was up-regulated from the Imd pathway, namely *relish* (GB13742), encoding a NF-κB-like transcription factor known to control the expression of *abaecin* and *Hymenoptaecin* in honeybees [58]. This was accompanied by down-regulation of two Imd pathway genes, *Tab* (GB18650) and *Tak1* (GB14664). These genes function in the regulation of the JNK pathway, which is believed to be involved in negative and positive feedback for AMP synthesis [59]. Three out of five genes were up-regulated from the JAK/STAT pathway, which is thought to contribute to immunity by inducing production of hemocytes and induction of complement-like factors [37]. However there was significant down-regulation of *NimC2* (GB13979). In *Drosophila*, Nimrod C1 (NimC1) is a protein component of the surface of hemocytes and is a determinant of phagocytic activity [60]. There was also significant down-regulation of a gene for an activator of prophenoloxidase, *PPOAct/SP8* (GB18767). The prophenoloxidase cascade is modulated by serine proteases and controls melanin synthesis, which is an important defense mechanism against invading extracellular pathogens including fungi [61]. Pathogens of other insects exhibit adaptations to counteracting phenoloxidase [62], [63]. Therefore this may be evidence of *M. anisopliae*-mediated inhibition of part of the honeybee immune response.

Our data also indicated that *M. anisopliae* infection of house bees affected the expression of genes involved in pathogen recognition acting upstream of the antimicrobial effector pathways. There was significant up-regulation of both of the known honeybee fibrinogen-related genes (*Angiopoietin*, GB17018; *Scabrous*, GB11902). In *Anopheles* and *Drosophila*, fibrinogen-related proteins function as pattern recognition receptors (PRRs) for activation of immune defenses against bacteria [64], [65]. Infection by *M. anisopliae* also resulted in significant up-regulation of the Gram-negative binding protein (GNBP) gene *B-gluc2* (GB19961). In termites, GNBP-2 functions both as a pattern recognition receptor of Gram-negative bacteria and fungi, including *M. anisopliae*, and as an antimicrobial effector protein [66]. In *Drosophila*, the presence of opportunistic fungal pathogens is detected by GNBP-3 operating upstream of the Toll pathway, but infection by entomopathogenic fungi is thought to directly activate Toll by cleavage of the *Drosophila* serine protease Persephone by the fungal protease Pr1 [67]. Our data also showed significant down-regulation of 4/14 genes encoding scavenger receptor (SCR) proteins (*AmSCR-B8*, GB16388; *AmSCR-B9*, GB19916; *AmSCR-B10*, GB19683; *AMSCR-C*, GB19925,). There was up-regulation of one C-lectin domain gene (*CTL2*, GB14265,). There was no differential expression of honeybee genes from the PRR immunoglobulin superfamily (IgSF). Insect IgSF proteins are present in the haemolymph and are assocated with binding to bacterial cells in the tobacco hornworm *Manduca sexta* (Lepidoptera) [68].

### Is the reduced susceptibility to infection in forager bees linked to up-regulation of candidate immune genes as a function of bee ageing?

Probably the most noticeable aspect of the microarray data was the effective absence of differential expression of candidate immune genes after treatment with *M. anisopliae* in forager bees compared to house bees. Only 2 genes were significantly differentially expressed in forager bees infected with *M. anisopliae*; down-regulation of *CTL2* (*C-type lectin 2*; GB14265) and up-regulation of IGFn3-2 (GB11358) a member of the immunoglobulin superfamily (IgSF). Can we link this finding with the observation that forager bees were less susceptible than house bees to the pathogen? Analysis of the microarray data for control bees (i.e. bees not treated with *M. anisopliae*) showed that foragers exhibited significant down-regulation of 6/12 honeybee C-type lectin genes compared to house bees. There was also significant down-regulation of 4/4 honeybee IgSF genes. C-type lectins function in aggregation reactions by binding hemocytes to microbial polysaccharides [69], while IgSF proteins are also associated with pathogen recognition and cell adhesion [70]. These observations are in keeping with published reports that hemocyte counts fall as honeybees age [28], [33]. Up-regulation of immunity related genes in foragers compared to house bees occurred in two areas. Firstly, there was significant up-regulation of the AMP gene *abaecin* (GB18323) alongside significant up-regulation of major gene components of the Toll pathway: *NEC-like* (GB16472, GB19582), *PSH–like/cSP14* (GB14044), *PSH-like/SP13* (GB15640), *Toll* (GB18520), *pelle* (GB16397), *cact-1* (GB10655) and *cact-2* (GB13520). Secondly, there was significant up-regulation of 12 genes encoding clip domain serine proteases (SPs) and serine-protease homologues (SPHs). These proteins, which occur in an evolutionarily diverse range of insects [71]–[73], are secreted into haemolymph as inactive zymogens and are components of cascade reactions that result in rapid activation of the Toll [73] and prophenoloxidase pathways [71], [74]. There was also significant up-regulation of three of the five honeybee serpin (Serine Protease Inhibitor) genes (*serpin-2*, GB16472; *serpin-3*. GB12279; and *serpin-5*, GB19582) which regulate the SP cascade and AMP synthesis [75]. The inference is that parts of the honeybee immune system were activated during the development of adult bees from the house to forager phenotype, resulting in greater resistance in foragers when they were subsequently treated with *M. anisopliae*. This may also account for the observation that only two immunity-related genes showed statistically significant differential expression in response to *M. anisopliae* in foragers. It is possible that immune system activation is part of the programmed development of the forager phenotype. This would be in keeping with other aspects of caste development in social insects which are associated with differential expression of shared genes, such as differentiation between honeybee queens and workers [76]. An alternative mechanism could be immune priming, a form of immune memory in which exposure to a pathogen results in reduced susceptibility upon later challenge [77], [78]. Adult honeybees are naturally exposed to fungal pathogens during their lives which could provide priming opportunities for long term protection. These pathogens include microsporidian fungi (*Nosema apis* and *Nosema ceranae* [3]) as well as ascomycete fungi, the most common being *Ascosphaera apis* (chalkbrood) and *Aspergillus flavus*, (stonebrood), although infections by other entomopathogenic ascomycete species including *Beauveria* and *Lecanicillium* have also been observed [6]. While it has not been demonstrated in all social insects [79], immune priming has been observed previously in the bumblebee *Bombus terrestris* [80] and in the unicolonial ant species *Lasius neglectus* [81]. Age-dependent effects on immunity have also been observed in *Drosophila*, with older flies showing increased expression of immune genes, and where variation in gene expression in different in-bred lines is linked to the ability to clear bacterial infection in older flies [82]. Up-regulation of *Drosophila* immune genes with age may be the result of pathogen exposure earlier in life [83], although there is also strong evidence of a decline in the ability to terminate AMP gene expression with age, resulting in a net increase in AMP production [84].

### Does host AMP synthesis have an adaptive benefit in the case of lethal infections?

Activation of the insect systemic immune response results in a time lag between host detection of pathogen elicitors and synthesis of AMPs. The systemic immune response is part of a complex, integrated system that also contains constitutive defenses to prevent invasion (for example, antifungal compounds on the cuticle [85]) as well as haemocytes that are responsible for rapid phagocytosis and nodulation reactions to restrain the development and survival of the pathogen early during invasion. This raises the question of the adaptive significance of AMPs, which come into play later in the infection process. One explanation is that AMPs evolved in insects as a system of clearing low level, persistent pathogens that had evaded constitutive/early acting defenses [86]. Clearly, in our study, strong up regulation of AMP synthesis in house bees failed to prevent lethal infection by *M. anisopliae*. However, AMP production during a lethal infection could be of adaptive benefit if it delays pathogen growth sufficiently to enable the host to increase its inclusive fitness by, for example, altruistic self-removal from the colony ( = adaptive suicide) [87]. Sick honeybees are known to engage in suicide behavior and modeling suggests that such self-removal from the colony to prevent transmission of pathogens should be commonplace in social insect species [88].

### Conclusions

The information provided in this study is a significant advance in developing our understanding of genome-wide honeybee defenses against pathogens. Experimental validation using loss of function studies will be required to confirm involvement of differentially expressed genes in the immune process. However, the system used here enables testable predictions to be made about the molecular mechanisms underlying the immune response. The study also provides evidence that immune capability does not decline in foragers, commensurate with the idea that bees exposed to pathogens at foraging sites are a route for introducing disease agents into the colony, providing a selection force for the maintenance of immunity [31], [32]. Our study focused primarily on the expression of genes associated with the honeybee humoral immune response, but it will be important in future to integrate this with information on other forms of defense, particularly the complex social responses of honeybees to pathogens [9], [11], [89].

The study of the honeybee immune system is of wide biological and practical interest. Numbers of *A. mellifera* colonies are declining in many regions of the world and this is causing considerable concern about the impact on crop production and the diversity of wild flowering plants [2]. Recent evidence has shown that pathogens are a key contributor to honeybee colony losses [2], [3], [4]. At present, the development of new interventions for disease management for beekeepers is being hampered by a lack of knowledge of the mechanisms of honeybee-pathogen interactions [4]. This is particularly the case at the molecular level. Our findings challenge previous assumptions that a strong innate immune response in honeybees is necessarily an indicator of greater resistance to infection in pathogens. It also provides further evidence of the importance of multi-level immunity operating in invertebrates.

## Materials and Methods

### Pathogen bioassay and survival analysis

A laboratory bioassay was used to quantify the susceptibility of known-age populations of adult *Apis mellifera* to *Metarhizium anisopliae* s.l. (Ascomycota, Hypocreales), a widespread generalist entomopathogenic fungus that has been used in a number of recent studies of host-pathogen interactions in social insects [91], [90]–[92] and which has also been used to study the molecular basis of the anti-fungal immune defense in *Drosophila* [67].

Honeybees were collected in summer (July) from a single colony, with a naturally mated queen, maintained in the apiary at Rothamsted Research, Harpenden UK. The Rothamsted colonies are typical to the UK in being a mixture of European subspecies and they are maintained according to conventional UK husbandry practice, which includes intensive treatment for varroa mites. None of the bees used in the experiment had symptoms of disease from naturally occurring pathogens, including honeybee viruses (e.g. physical deformities, unusual movement), and none of the bees were observed to harbor phoretic varroa mites. Bees were treated with *M. anisopliae* s.l. strain 445.99 ( = the strain code used in the Warwick University collection of entomopathogenic fungus cultures). This strain is used as the active ingredient of the commercial mycoinsecticide Bio-Blast (Eco-Science Corp. USA) developed as a biological control agent of termites [93]. Conidia powder was collected from cultures of *M. anisopliae* 445.99 grown on Sabouraud dextrose agar for 10 days at 22°C and was passed through a 250 µm sieve.

The bioassay comprised two cohorts of honeybees of known ages. For cohort 1, brood frames containing pupae were removed from the colony to an observation chamber in an incubator (34°C) 26 days before the bioassay. Approximately 1000 adult worker bees that emerged over a 24 hr period were marked on the thorax using modelling paint, and then returned to the colony. The evening before the bioassay, a mesh field cage (3×3×2 m) was placed over the colony to confine foragers emerging from the colony the next morning. Approximately 200 marked bees were then collected as foragers and placed individually in bijou bottles within an insulated cooler box. Cohort 2 consisted of one day old adult bees collected from a brood frame from the same colony and held in an observation chamber as described above.

For each cohort, groups of 15 honeybees were placed in Universal bottles containing 0.5 g of *M. anisopliae* conidia powder. Controls were placed in bottles with no conidia powder. Bottles were rotated gently for 30 s and then left at 30°C in darkness for 30 min to give time for honeybees to shake off excess powder. Each group of 15 honeybees was then transferred to a clear Perspex box (13 cm×4 cm×4 cm and drilled with ventilation holes) lined with a sheet of tissue paper and containing two drip feeders (one with distilled water and one with 10% sucrose solution). Boxes were maintained in darkness at 30°C and 72% RH for 24 hrs before being maintained at ambient humidity for the remainder of the bioassay. Water and sucrose feed solution were changed *ad libitum*.

A census of survivorship was done twice a day for six days. All groups of honeybees were handled in the same way. Dead honeybees were incubated on damp filter paper within Petri dishes and observed for the presence of sporulating mycelium of *M. anisopliae* in order to confirm fungus-induced mortality. A small number of honeybees found dead less than 12 hrs after treatment were assumed to have died as a result of handling and were removed from the experiment. Controls consisted of two batches of 15 honeybees each (*n* = 30), and fungus-treatments consisted of four batches of 15 honeybees each (*n* = 60). In addition, one extra bioassay box was set up for each of the four treatments. After 48 hrs, honeybees from these boxes were transferred to liquid nitrogen and then stored at −80°C prior to RNA extraction (this time was chosen as it takes at c. 48 h for spores of *M. anisopliae* s.l. to germinate on an insect surface and then penetrate into the body [94]).

We tested for differences in survival between each of the four experimental treatments, (house honeybees, forager honeybees)×(uninfected, infected), using parametric survival regression [95]. Groups of biological replicates were modelled as gamma distributed random effects [96].

### RNA extraction and probe preparation

Individual honeybees were ground in liquid nitrogen. RNA extraction was done on 50 mg powdered material using TRIzol Reagent (Invitrogen) according to the manufacturer's instructions. Total RNA was purified using RNeasy spin columns (Qiagen RNeasy Plant Mini kit) and treated with RNAse-free DNAse I (New England Biolabs). RNA concentration and purity was determined by lab-on-chip analysis using a 2100 Bioanalyzer and an RNA 6000 LabChip (Agilent Technologies). 1 µg of total RNA from each total RNA preparation from an individual honeybee was amplified to produce Cy3- or Cy5-labelled aRNA probes using a low input RNA fluorescent linear amplification kit (Agilent Technologies, Santa Clara USA).

### Microarray transcriptional profiling

The eArray platform from Agilent Technologies was used to design 60-mer oligonucleotide probes for a microarray based on the *A. mellifera* transcriptome, comprising 10498 mRNA sequences from the Official Honeybee Gene Set 1 [97]. In addition, 22 sequences from eight honeybee viruses taken from GenBank were included: deformed wing virus (DWV); *Varroa destructor* virus (VDV-1); honeybee slow paralysis virus; black queen cell virus; acute bee paralysis virus; Kashmir bee virus; Israeli acute paralysis virus; and sacbrood virus. The microarray slide (Agilent Design ID: 019875) consisted of eight arrays of 15000 elements each including honeybee and virus probes as well as standard internal controls.

### Microarray experiment design

The microarray experiment used a two-channel (dye) system to make direct comparisons between pairs of samples within a customised Agilent 8-pack array (with each slide containing eight separate arrays, and with each array having two independent samples applied, one labelled with each of the two dyes). Four slides were available for the experiment, providing 32 arrays to make comparisons between the 32 samples included in the experiment. These 32 samples comprised eight biological replicates of each of four treatment combinations – forager honeybees treated with *M. anisopliae*, forager honeybees not treated with *M. anisopliae*, house honeybees treated with *M. anisopliae*, house honeybees not treated with *M. anisopliae* – considered to comprise a 2-by-2 factorial structure for honeybee type (forager, house) and infection status (infected with *M. anisopliae*, uninfected). Each of the 32 samples was hybridised to two different arrays, once with each dye, and was co-hybridised with two different other samples, as follows:

- Each infected forager honeybee sample was co-hybridised with an infected house honeybee sample on one array and with an uninfected forager honeybee sample on a second array.
- Each infected house honeybee sample was co-hybridised with an uninfected house honeybee sample on one array and with an infected forager honeybee sample on a second array.
- Each uninfected house honeybee sample was co-hybridised with an uninfected forager honeybee sample on one array and with an infected house honeybee sample on a second array.
- Each uninfected forager honeybee sample was co-hybridised with an infected forager honeybee sample on one array and with an uninfected house honeybee sample on a second array.

Each slide contained two arrays for each of the four possible treatment comparisons, with most comparisons within an array being between samples given the same arbitrary biological replicate labels, but with all direct comparisons between uninfected forager honeybee samples and infected forager honeybee samples being between differently labelled biological replicates (see Figure S2 for a full diagrammatic representation of this design). This linking of the arbitrarily labelled biological replicates ensured that the design was fully connected (each sample can be indirectly compared with every other sample), also providing links between the observations made on different slides.

### Microarray analysis

Microarray slide scanning was done using an Agilent Technologies GA2565BA Scanner. Microarray data were processed from raw data image files using feature extraction software (Agilent Technologies). At each probe location, Cy3 and Cy5 intensities were measured as median values of green and red pixels respectively. All probe measurements were corrected for local background intensities. In addition, dark corner corrections were made for each array. Preliminary data inspection supported normalisation by logarithm transformation; base two allowed for intuitive interpretation of changes in gene regulation (a difference of one equates to a two fold change in expression). Spatial bias across arrays was controlled with two-dimensional local smoothing (‘loess’) separately for each array. This processed dataset was used to test hypotheses on the effects of honeybee role and infection status on whole genome expression. Statistical analyses were conducted using the R statistical programming platform, version 2.7.1 (http://www.r-project.org). Processed data were modelled in a mixed effects framework using the MAANOVA library from the bioconductor suite of packages (http://www.bioconductor.org, accessed 03/07/12).

Consistent with our bioassay, we used a factorial experimental design, (house honeybee, forager honeybee)×(uninfected, infected with *M. anisopliae*). We were motivated to understand transitions between age-related stages (house→forager) and fungal disease states (uninfected→infected), quantifying the appropriate contrasts: uninfected *vs.* infected house honeybees; uninfected *vs.* infected forager honeybees; and uninfected house honeybees *vs.* uninfected forager honeybees. Since fungal infected house honeybees died before developing into forager honeybees, this final contrast was not explicitly quantified. In addition, we modelled the presence of naturally occurring asymptomatic viruses within sampled honeybees as a two level (‘low’, ‘high’) categorical covariate (see supplementary information Figure S3). We modelled other experimental sources of uncertainty as variance components (‘slide’ crossed with ‘array’, and ‘dye’). Two level experimental treatment contrasts were assessed using *t*-tests. We identified changes in expression with a probability threshold of *p*<(1/number of probes), thereby reducing the expected false positives to less than one probe [98]. Changes in expression identified as significant were further categorised as up- or down-regulated. The set of raw microarray data is available via ArrayExpress at the European Bioinformatics Institute (accession number E-MTAB-1214).

### Bioinformatics analysis

The microarray statistical analysis identified sets of genes that were differentially expressed in association with the treatment contrasts used in the bioassay. These sets of differentially expressed genes were subject to Gene Ontology (GO) analysis to identify significantly over-represented GO terms. As functional annotation of the bee genome is incomplete, we ascribed putative Gene Ontology classifications to as many genes as possible based on homology to *Drosophila melanogaster*. Using reciprocal best-BLAST hit (RBH) criteria, 6325 (62%) of honeybee genes had an assignable fly ortholog. We were then able to determine which GO categories are statistically over-represented in groups of differentially expressed genes, using Cytoscape (version 2.6.0, Agilent Technologies) and the BiNGO Plug-in. Over-representation of terms was determined through a Hypergeometric test (0.05 significance level), using the Venn diagram intersection combination genes versus the whole genome annotation as the background ‘universe’. Benjamini & Hochberg False Discovery Rate correction was applied. FlyBase gene identifiers were converted to EntrezGene IDs using Ensembl Biomart via the webserver (http://www.ensembl.org).

### qRT-PCR analysis

The expression of honeybee beta actin and vitellogenin genes, as well as the levels of *M. anisopliae* rRNA, were analysed using qRT-PCR for each of the 32 biological replicates in the experiment. Superscript II reverse transcriptase (Invitrogen) and random hexanucleotides were used to produce cDNA from DNAse I treated total RNA. Real time quantitative PCR was carried out using the Platinum SYBR Green qPCR kit (Invitrogen) in triplicates in 20 µL reactions in the ABI PRISM 7900HT system (Applied Biosystems). The amplification program included 2 min at 50°C, 10 min at 95°C, and 40 cycles, 95°C for 15 sec, 60°C for 1 min. Honeybee beta actin mRNA was quantified using primers 5′-AGGAATGGAAGCTTGCGGTA-3′ and 5′-AATTTTCATGGTGGATGGTGC-3′. Honeybee vitellogenin mRNA was quantified using primers 5′-cggcACGAGTACCTGGACAAGGCcG-3′ and 5′-TCCTTGAAATGTGCATCCATGA -3′. Finally, *M. anisopliae* 18 s rRNA was quantified with the primers 5′-CCAACCCCTGTGAATTATACC-3′ and 5′-CGATCCCCAACACCAAGTC-3′.

## Supporting Information

Figure S1 **Box-Whisker plots of RT-PCR quantification for honeybee** ***actin*** **,** ***vitellogenin*** **and** ***M. anisopliae*** **s.l mRNA.** Expression levels for each of the four experimental treatments: uninfected house honeybees; house honeybee infected with *M. anisopliae*; uninfected forager honeybees; and forager honeybees infected with *M. anisopliae* (*n* = 8 for each treatment group). Boxes denote interquartile range, bisected horizontally by median values; whiskers extend to 1.5× interquartile range beyond boxes; outliers are marked as dots beyond whiskers. Expression is shown as the inverse of number of amplification cycles to reach Critical Threshold values (C_T_^−1^).
(PDF)

Figure S2 **Design for microarray experiment.** Treatment codes indicate forager (F) or house (H) honeybee, infected (I) or uninfected (U) with *M. anisopliae* s.l., and biological replicate (arbitrary label 1–8). Arrows join samples compared on the same array, with the pointed end of the arrow indicating one dye and the blunt end the other dye. Arrays with the same colour arrow were included on the same slide.
(PDF)

Figure S3 **Pairwise correlation plots of abundance for honeybee viruses detected by microarray.** The associated bar chart indicates variance in RNA virus levels partitioned into four orthogonal principal components. Horizontal dotted line denotes mean variance – Kaiser's criterion – with PC1 the only principle component to exceed this value (suggesting the first principle component is adequate to explain variation).
(PDF)

Figure S4 **Cluster analysis of honeybee virus abundance data.** Datapoints mark (PC1, PC2) coordinates of virus expression levels from all honeybee samples in our microarray experiment. Dotted lines show density of datapoints on each axis. K-means analysis indicated two clusters, centred on points marked “+”. These two virus expression clusters were explained using hierarchical recursive partitioning of deviance, including honeybee role (house, forager), fungal treatment (uninfected, infected with *M. anisopliae*), as well as experimental sources of variance: slide, array and dye. Trees indicate observed deviance was explained primarily by honeybee role and secondarily by infection status. Experimental sources of variance did not explain significant amounts of observed deviance. We concluded this analysis by designating individual honeybees as having either ‘high’ or ‘low’ levels of virus.
(PDF)

Table S1 **GO terms (Level 3,** ***Drosophila melanogaster*** **) of differentially-expressed genes (** http://genecodis.dacya.ucm.es/analysis/ **) associated with** ***D. melanogaster*** **orthologs of** ***Apis mellifera*** **genes.** Only the Level 3 terms with *P*<0.05 are shown.
(PDF)

Table S2 **Statistically significant (** ***p*** **<1/** ***n*** **) differences in expression of immune related genes (based on previous homology assignments** [**34**] **) between house and forager honeybees in response to treatment with** ***M. anisopliae*** **s.l.** Numbers in table columns refer to fold change (log_2_) in gene expression.
(PDF)
