# Supplementary material for: A Strong Immune Response in Young Adult Honeybees Masks Their Increased Susceptibility to Infection Compared to Older Bees
Source: PLoS Pathog. 2012 Dec 27;8(12):e1003083. doi: 10.1371/journal.ppat.1003083 (PMC3531495; doi:10.1371/journal.ppat.1003083)
Supplement: Figure S1 — Box-Whisker plots of RT-PCR quantification for honeybee actin , vitellogenin and M. anisopliae s.l mRNA. Expression levels for each of the four experimental treatments: uninfected house honeybees; house honeybee infected with M. anisopliae; uninfected forager honeybees; and forager honeybees infected with M. anisopliae (n = 8 for each treatment group). Boxes denote interquartile range, bisected horizontally by median values; whiskers extend to 1.5× interquartile range beyond boxes; outliers are marked as dots beyond whiskers. Expression is shown as the inverse of number of amplification cycles to reach Critical Threshold values (CT −1). (PDF) [file ppat.1003083.s001.pdf]

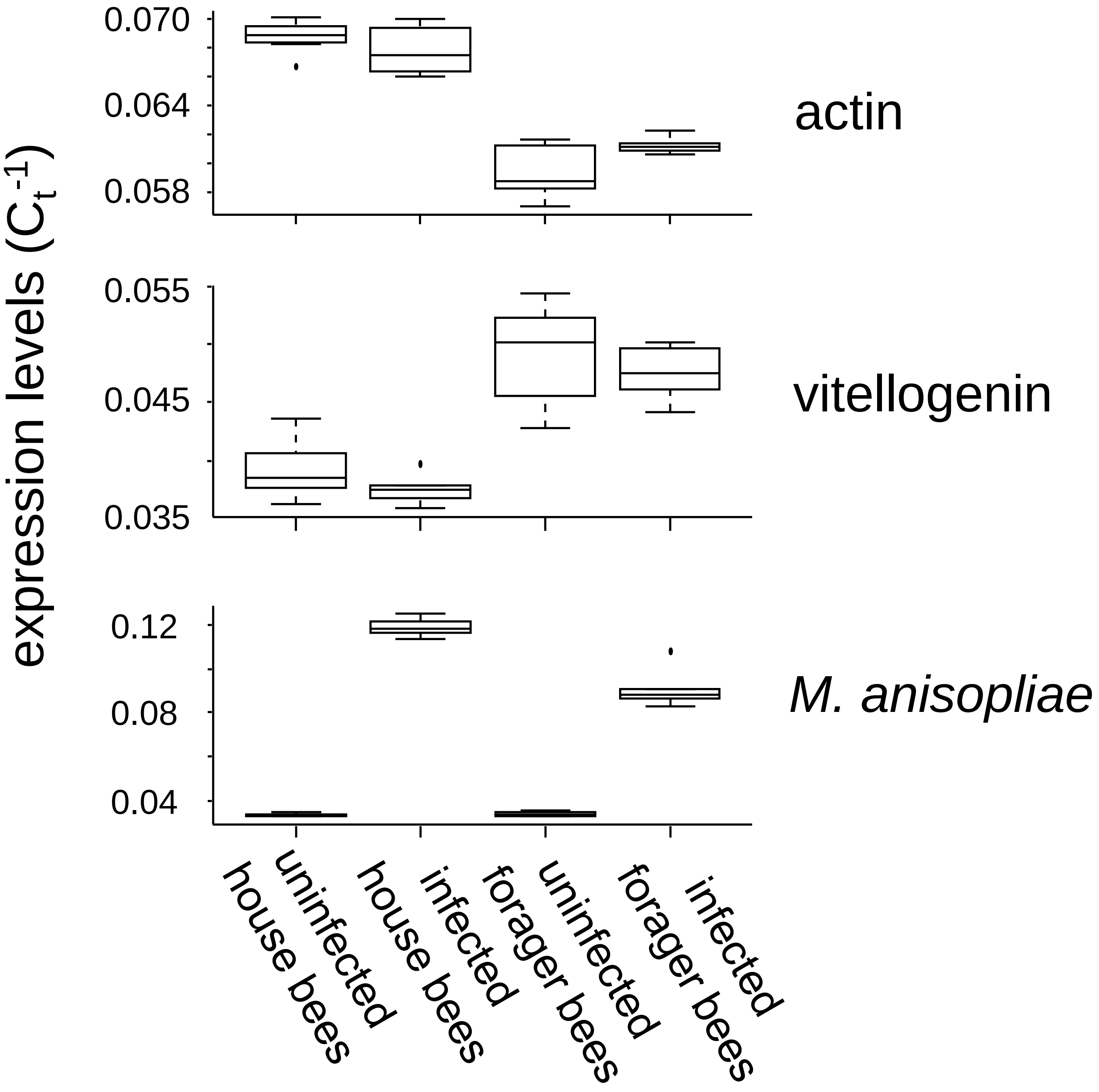

expression levels (C_t^{-1})
0.070
0.064
0.058
actin
0.055
0.045
0.035
vitellogenin
0.12
0.08
0.04
M. anisopliae
house bees uninfected
house bees infected
forager bees uninfected
forager bees infected
