# Supplementary figures and images for: A Strong Immune Response in Young Adult Honeybees Masks Their Increased Susceptibility to Infection Compared to Older Bees
Source: PLoS Pathog. 2012 Dec 27;8(12):e1003083. doi: 10.1371/journal.ppat.1003083 (PMC3531495; doi:10.1371/journal.ppat.1003083)

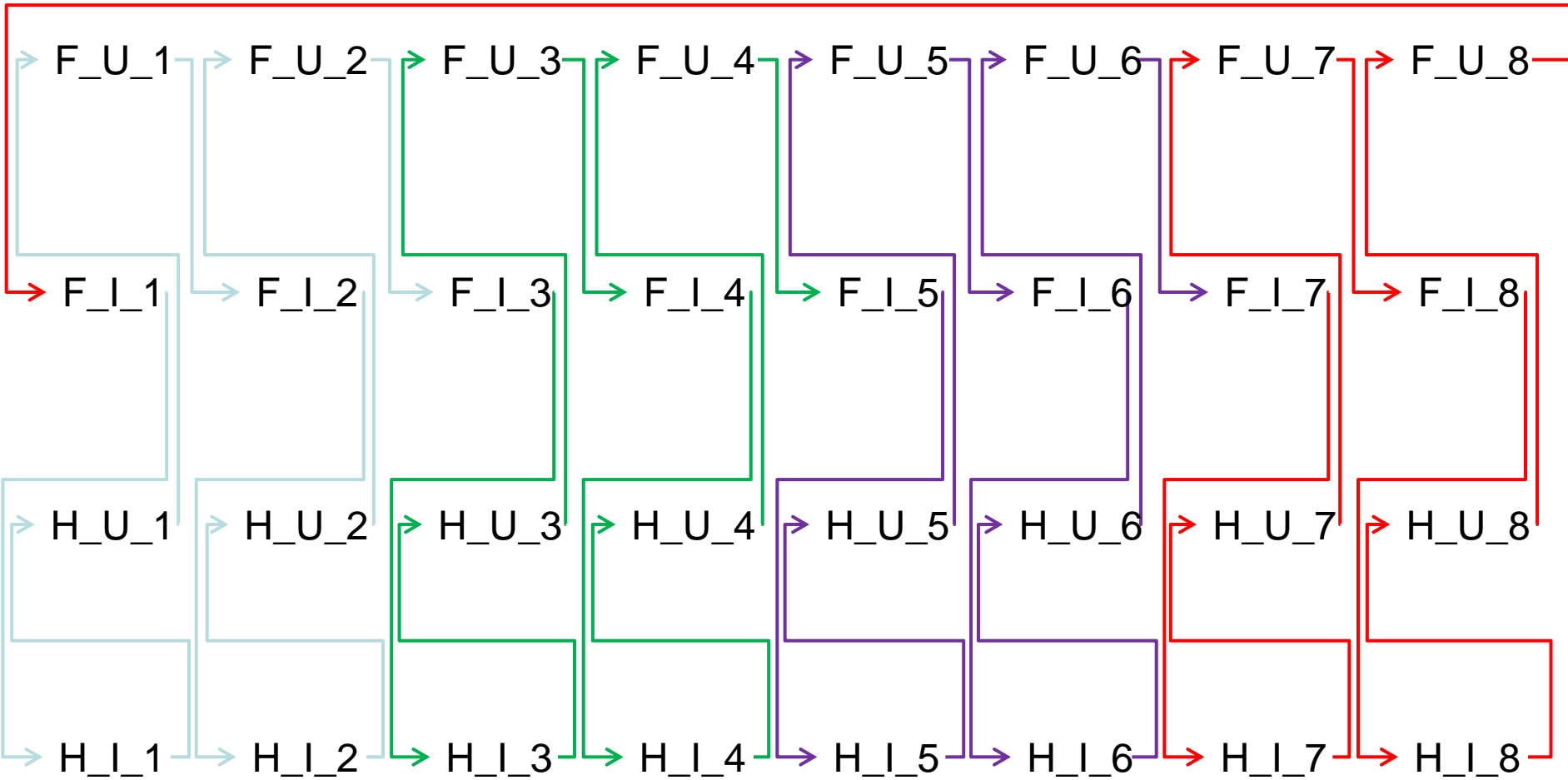

Supplement: Figure S2 — Design for microarray experiment. Treatment codes indicate forager (F) or house (H) honeybee, infected (I) or uninfected (U) with M. anisopliae s.l., and biological replicate (arbitrary label 1–8). Arrows join samples compared on the same array, with the pointed end of the arrow indicating one dye and the blunt end the other dye. Arrays with the same colour arrow were included on the same slide. (PDF) [file ppat.1003083.s002.pdf]

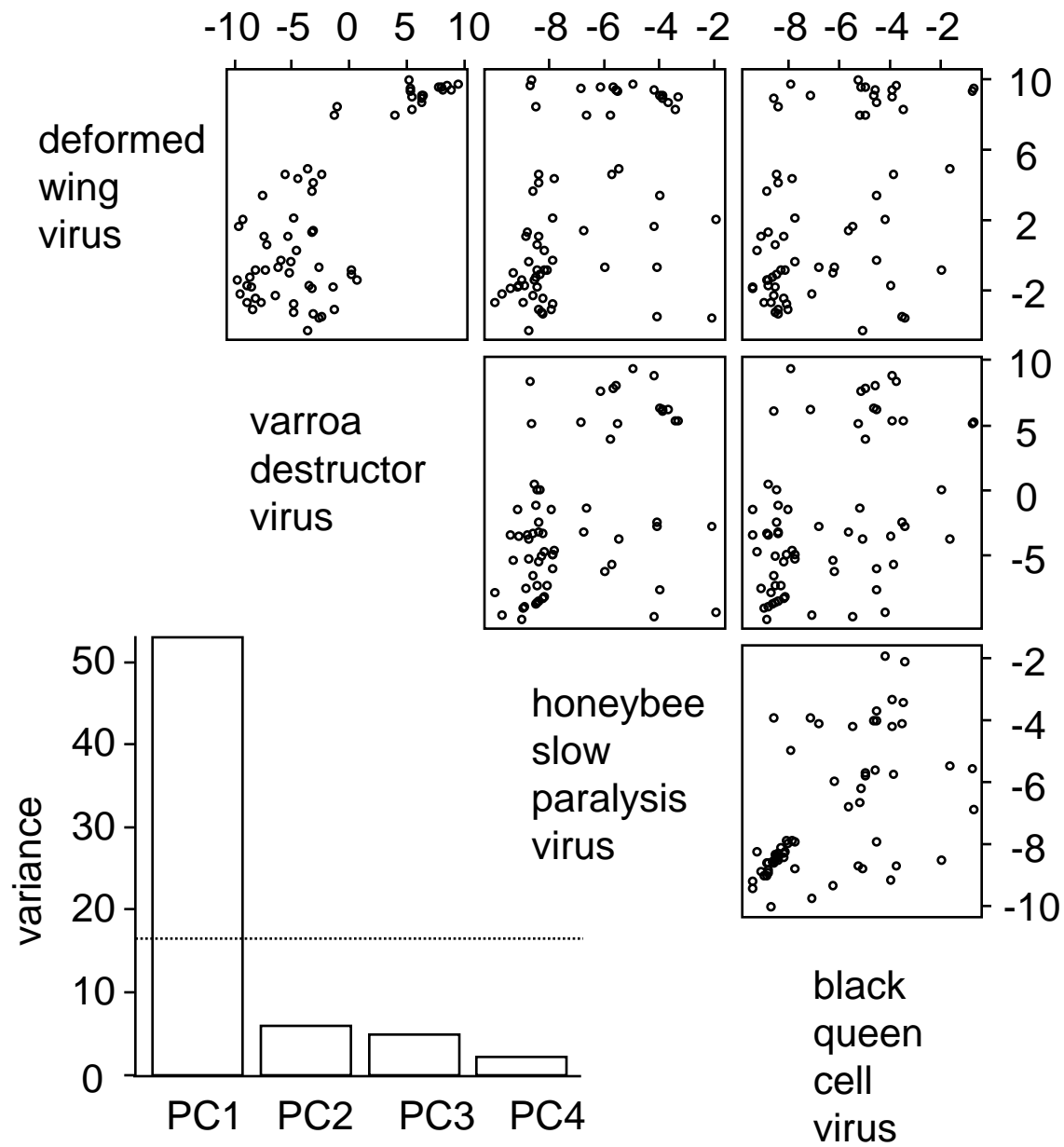

Supplement: Figure S3 — Pairwise correlation plots of abundance for honeybee viruses detected by microarray. The associated bar chart indicates variance in RNA virus levels partitioned into four orthogonal principal components. Horizontal dotted line denotes mean variance – Kaiser's criterion – with PC1 the only principle component to exceed this value (suggesting the first principle component is adequate to explain variation). (PDF) [file ppat.1003083.s003.pdf]

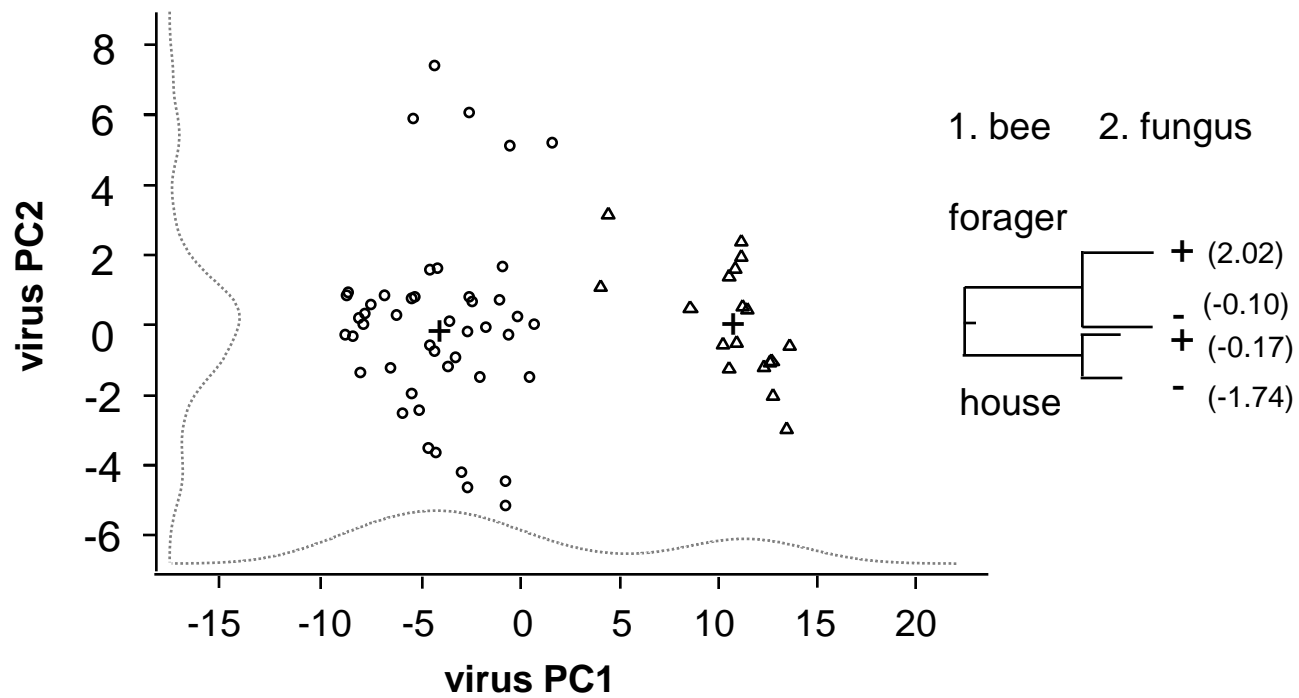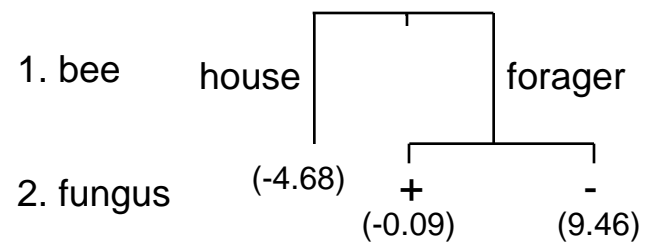

Supplement: Figure S4 — Cluster analysis of honeybee virus abundance data. Datapoints mark (PC1, PC2) coordinates of virus expression levels from all honeybee samples in our microarray experiment. Dotted lines show density of datapoints on each axis. K-means analysis indicated two clusters, centred on points marked “+”. These two virus expression clusters were explained using hierarchical recursive partitioning of deviance, including honeybee role (house, forager), fungal treatment (uninfected, infected with M. anisopliae), as well as experimental sources of variance: slide, array and dye. Trees indicate observed deviance was explained primarily by honeybee role and secondarily by infection status. Experimental sources of variance did not explain significant amounts of observed deviance. We concluded this analysis by designating individual honeybees as having either ‘high’ or ‘low’ levels of virus. (PDF) [file ppat.1003083.s004.pdf]
