# Supplementary material for: A Strong Immune Response in Young Adult Honeybees Masks Their Increased Susceptibility to Infection Compared to Older Bees
Source: PLoS Pathog. 2012 Dec 27;8(12):e1003083. doi: 10.1371/journal.ppat.1003083 (PMC3531495; doi:10.1371/journal.ppat.1003083)
Supplement: Table S1 — GO terms (Level 3, Drosophila melanogaster ) of differentially-expressed genes ( http://genecodis.dacya.ucm.es/analysis/ ) associated with D. melanogaster orthologs of Apis mellifera genes. Only the Level 3 terms with P<0.05 are shown. (PDF) [file ppat.1003083.s005.pdf]

Table S1. GO terms (Level 3, *Drosophila melanogaster*) of differentially-expressed genes (http://genecodis.dacya.ucm.es/analysis/) associated with *D. melanogaster* orthologs of *Apis mellifera* genes. Only the Level 3 terms with $P < 0.05$ are shown. Letters in the first column ("cluster") refer to the Venn diagram sectors in Figure 2. Cluster "c+e+f+g" represents genes that were differentially expressed with bee development (house bees → forager bees); cluster "a+e" represents genes that were differentially expressed in house bees in response to *M. anisopliae* infection but not in forager bees. BP = biological process. MF = molecular function. CC = cellular component.

cluster	GO ID (category)	description	p-value
"c+e+f+g"	GO:6091 (BP)	generation of precursor metabolites and energy	3.16E-33
"c+e+f+g"	GO:8151 (BP)	cellular process	1.49E-24
"c+e+f+g"	GO:6119 (BP)	oxidative phosphorylation	1.49E-24
"c+e+f+g"	GO:22900 (BP)	electron transport chain	1.51E-22
"c+e+f+g"	GO:55114 (BP)	oxidation reduction	2.64E-22
"c+e+f+g"	GO:22904 (BP)	respiratory electron transport chain	6.05E-22
"c+e+f+g"	GO:42773 (BP)	ATP synthesis coupled electron transport	5.03E-20
"c+e+f+g"	GO:42775 (BP)	organelle ATP synthesis coupled electron transport	8.48E-18
"c+e+f+g"	GO:44237 (BP)	cellular metabolic process	2.41E-17
"c+e+f+g"	GO:6333 (BP)	chromatin assembly or disassembly	4.20E-16
"c+e+f+g"	GO:8152 (BP)	metabolic process	6.55E-16
"c+e+f+g"	GO:6325 (BP)	establishment/maintenance of chromatin architecture	3.46E-14
"c+e+f+g"	GO:6120 (BP)	mitochondrial NADH to ubiquinone	4.52E-11
"c+e+f+g"	GO:7001 (BP)	chromosome organization and biogenesis	2.08E-10
"c+e+f+g"	GO:6796 (BP)	phosphate metabolic process	4.92E-09
"c+e+f+g"	GO:6793 (BP)	phosphorus metabolic process	4.92E-09
"c+e+f+g"	GO:16310 (BP)	phosphorylation	7.79E-09
"c+e+f+g"	GO:6996 (BP)	organelle organization and biogenesis	7.51E-08
"c+e+f+g"	GO:44238 (BP)	primary metabolic process	2.92E-07
"c+e+f+g"	GO:15980 (BP)	energy derivation by oxidation of organic compounds	1.04E-06
"c+e+f+g"	GO:51186 (BP)	cofactor metabolic process	1.54E-06
"c+e+f+g"	GO:16043 (BP)	cellular component organization and biogenesis	1.64E-06
"c+e+f+g"	GO:16651 (MF)	oxidoreductase activity, acting on NADH or NADPH	3.14E-14
"c+e+f+g"	GO:3954 (MF)	NADH dehydrogenase activity	3.14E-14
"c+e+f+g"	GO:50136 (MF)	NADH dehydrogenase (quinone) activity	2.24E-11
"c+e+f+g"	GO:8137 (MF)	NADH dehydrogenase (ubiquinone) activity	2.24E-11
"c+e+f+g"	GO:16655 (MF)	oxidoreductase activity	2.24E-11
"c+e+f+g"	GO:3824 (MF)	catalytic activity	3.58E-11
"c+e+f+g"	GO:16491 (MF)	oxidoreductase activity	1.48E-08
"c+e+f+g"	GO:3740 (MF)	structural constituent of ribosome	2.77E-08
"c+e+f+g"	GO:15078 (MF)	hydrogen ion transmembrane transporter activity	4.36E-07
"c+e+f+g"	GO:15077 (MF)	inorganic cation transmembrane transporter	6.67E-07
"c+e+f+g"	GO:22890 (MF)	inorganic cation transmembrane transporter	7.57E-06
"c+e+f+g"	GO:32991 (CC)	macromolecular complex	1.59E-39

"c+e+f+g"	GO:786 (CC)	nucleosome	7.28E-39
"c+e+f+g"	GO:44446 (CC)	intracellular organelle part	6.80E-37
"c+e+f+g"	GO:44422 (CC)	organelle part	7.67E-37
"c+e+f+g"	GO:44424 (CC)	intracellular part	9.09E-35
"c+e+f+g"	GO:32993 (CC)	protein-DNA complex	1.12E-34
"c+e+f+g"	GO:5622 (CC)	intracellular	8.33E-34
"c+e+f+g"	GO:5623 (CC)	cell	1.68E-32
"c+e+f+g"	GO:44464 (CC)	cell part	1.68E-32
"c+e+f+g"	GO:43229 (CC)	intracellular organelle	1.59E-28
"c+e+f+g"	GO:43226 (CC)	organelle	1.74E-28
"c+e+f+g"	GO:5717 (CC)	chromatin	7.67E-28
"c+e+f+g"	GO:44444 (CC)	cytoplasmic part	1.12E-27
"c+e+f+g"	GO:5737 (CC)	cytoplasm	5.75E-27
"c+e+f+g"	GO:44455 (CC)	mitochondrial membrane part	5.75E-26
"c+e+f+g"	GO:44429 (CC)	mitochondrial part	7.17E-25
"c+e+f+g"	GO:5740 (CC)	mitochondrial envelope	4.15E-23
"c+e+f+g"	GO:31966 (CC)	mitochondrial membrane	3.61E-22
"c+e+f+g"	GO:5746 (CC)	mitochondrial respiratory chain	5.68E-22
"c+e+f+g"	GO:5739 (CC)	mitochondrion	8.28E-22
"c+e+f+g"	GO:5743 (CC)	mitochondrial inner membrane	8.30E-22
"c+e+f+g"	GO:31967 (CC)	organelle envelope	1.59E-21
"c+e+f+g"	GO:31975 (CC)	envelope	1.98E-21
"c+e+f+g"	GO:19866 (CC)	organelle inner membrane	8.70E-21
"c+e+f+g"	GO:31090 (CC)	organelle membrane	1.31E-19
"c+e+f+g"	GO:44427 (CC)	chromosomal part	3.25E-19
"c+e+f+g"	GO:5694 (CC)	chromosome	6.01E-19
"c+e+f+g"	GO:5811 (CC)	lipid particle	1.15E-17
"c+e+f+g"	GO:43232 (CC)	intracellular non-membrane-bounded organelle	9.24E-16
"c+e+f+g"	GO:43228 (CC)	non-membrane-bounded organelle	9.24E-16
"c+e+f+g"	GO:5747 (CC)	mitochondrial respiratory chain complex I	5.48E-15
"c+e+f+g"	GO:45271 (CC)	respiratory chain complex I	5.48E-15
"c+e+f+g"	GO:30964 (CC)	NADH dehydrogenase complex	5.48E-15
"c+e+f+g"	GO:43234 (CC)	protein complex	1.64E-12
"c+e+f+g"	GO:43231 (CC)	intracellular membrane-bounded organelle	1.93E-09
"c+e+f+g"	GO:43227 (CC)	membrane-bounded organelle	2.21E-09
"c+e+f+g"	GO:5840 (CC)	ribosome	2.36E-09
"c+e+f+g"	GO:5830 (CC)	cytosolic ribosome	1.05E-08
"c+e+f+g"	GO:33279 (CC)	ribosomal subunit	2.15E-08
"c+e+f+g"	GO:44445 (CC)	cytosolic part	3.30E-07
"c+e+f+g"	GO:45259 (CC)	proton-transporting ATP synthase complex	3.25E-06
"c+e+f+g"	GO:5753 (CC)	mitochondrial H+ transporting ATP synthase complex	3.25E-06
"c+e+f+g"	GO:30529 (CC)	ribonucleoprotein complex	5.10E-06
"a+e"	GO:16043 (BP)	cellular component organization and biogenesis	6.54E-06
"a+e"	GO:65007 (BP)	biological regulation	6.54E-06
"a+e"	GO:6996 (BP)	organelle organization and biogenesis	6.54E-06
"a+e"	GO:50791 (BP)	regulation of biological process	6.54E-06
"a+e"	GO:5488 (MF)	binding	5.64E-10
"a+e"	GO:3676 (MF)	nucleic acid binding	1.13E-06
"a+e"	GO:5623 (CC)	cell	3.02E-17
"a+e"	GO:44464 (CC)	cell part	3.02E-17
"a+e"	GO:5622 (CC)	intracellular	4.90E-15
"a+e"	GO:44424 (CC)	intracellular part	2.83E-12
"a+e"	GO:5830 (CC)	cytosolic ribosome	5.99E-12

"a+e"	GO:44445 (CC)	cytosolic part	6.39E-10
"a+e"	GO:43229 (CC)	intracellular organelle	7.08E-10
"a+e"	GO:43226 (CC)	organelle	7.08E-10
"a+e"	GO:43232 (CC)	intracellular non-membrane-bounded organelle	9.39E-10
"a+e"	GO:43228 (CC)	non-membrane-bounded organelle	9.39E-10
"a+e"	GO:786 (CC)	nucleosome	2.68E-08
"a+e"	GO:32993 (CC)	protein-DNA complex	3.79E-08
"a+e"	GO:30873 (CC)	cytosolic small ribosomal subunit	1.53E-06
"a+e"	GO:5634 (CC)	nucleus	1.53E-06
"a+e"	GO:33279 (CC)	ribosomal subunit	3.11E-06
"a+e"	GO:5717 (CC)	chromatin	3.75E-06
"a+e"	GO:30498 (CC)	cytosolic large ribosomal subunit	5.47E-06
"a+e"	GO:44446 (CC)	intracellular organelle part	8.78E-06
"a+e"	GO:44422 (CC)	organelle part	9.23E-06
