# Supplementary material for: A Strong Immune Response in Young Adult Honeybees Masks Their Increased Susceptibility to Infection Compared to Older Bees
Source: PLoS Pathog. 2012 Dec 27;8(12):e1003083. doi: 10.1371/journal.ppat.1003083 (PMC3531495; doi:10.1371/journal.ppat.1003083)
Supplement: Table S2 — Statistically significant ( p <1/ n ) differences in expression of immune related genes (based on previous homology assignments [34] ) between house and forager honeybees in response to treatment with M. anisopliae s.l. Numbers in table columns refer to fold change (log2) in gene expression. (PDF) [file ppat.1003083.s006.pdf]

Table S2: Statistically significant ($p < 1/n$) differential expression of immune related genes (based on previous homology assignments [34] in house and forager honeybees treated with *M. anisopliae* s.l. Numbers in columns refer to fold change (log_2) in gene expression.

**Gene name**	**Official name**	**Gene family / pathway**	**House bees**	**Forager bees**	**Controls (uninfected bees)**
			**uninfected → infected**	**uninfected → infected**	**House → forager**
abaecin	GB18323	AMP	**4.13**		**3.68**
Defensin-2	GB10036	AMP	**4.97**		
Hymenoptaecin	GB17538	AMP	**6.39**		
Hemolectin	GB16711	Cellular response	**-0.44**		
Hemomucin	GB20003	Cellular response			**0.55**
CTL1	GB11717	C-lectin domain			**-2.18**
CTL2	GB14265	C-lectin domain	**0.54**	**-0.47**	**0.98**
CTL3	GB18049	C-lectin domain			**-2.27**
CTL4	GB20122	C-lectin domain			**-2.11**
CTL6	GB11792	C-lectin domain			**-1.25**
CTL7	GB14975	C-lectin domain			**-1.84**
CTL9	GB15050	C-lectin domain			**-2.36**
Angiopoietin	GB17018	Fibrinogen	**0.61**		**-1.28**
Scabrous	GB11902	Fibrinogen	**0.37**		**0.67**
B-gluc2	GB19961	GNBPs	**1.91**		**3.28**
IGFn3-11	GB12490	IG Superfamily Genes			**-1.51**
IGFn3-15	GB15344	IG Superfamily Genes			**-0.41**
IGFn3-2	GB11358	IG Superfamily Genes		**0.52**	**-1.49**
IGFn3-7	GB11846	IG Superfamily Genes			**-0.60**
dUbc13	GB19498	IMD			**0.49**
lap2	GB11057	IMD			**0.48**
relish	GB13742	IMD	**0.58**		
Tab	GB18650	IMD	**-0.29**		
Tak1	GB14664	IMD	**-0.48**		
Domeless	GB12159	JakSTAT	**0.91**		
D-PIAS	GB18362	JakSTAT	**0.28**		**0.46**
SOCS	GB18949	JakSTAT	**0.23**		
RIP1	GB11320	MAPK	**0.50**		
NimA	GB12883	Phagocytosis			**-0.88**
NimB	GB12454	Phagocytosis			**1.24**
NimC2	GB13979	Phagocytosis	**-1.18**		
PGRP-S1	GB15371	PGRP	**-1.22**		**1.23**
PGRP-S2	GB19301	PGRP	**3.43**		**2.26**
PGRP-S3	GB17879	PGRP	**-0.88**		**2.36**
PPO	GB18313	PPO			**-1.76**

PPOAct / SP8	GB18767	PPO / serine protease	**-1.70**		
GRAAL/Tequila-like / SP23	GB12538	Scav. Receptor A / serine protease			**1.23**
AmSCR-B10	GB19683	Scav. Receptor B	**-0.63**		
AmSCR-B8	GB16388	Scav. Receptor B	**-0.73**		
AmSCR-B9	GB19916	Scav. Receptor B	**-0.69**		**0.67**
AmSCR-C	GB19925	Scav. Receptor C	**-1.68**		
cSP1	GB16147	serine proteases			**1.30**
cSP2	GB14247	serine proteases			**1.71**
cSP7	GB17145	serine proteases			**2.13**
cSPH42	GB11298	serine proteases			**1.26**
SP16	GB12253	serine proteases	**0.59**		
SP23	GB12538	serine proteases			**1.23**
SP24	GB14233	serine proteases			**-0.68**
SP28	GB13489	serine proteases			**2.66**
SP34	GB11552	serine proteases			**-5.71**
SP35	GB16021	serine proteases			**2.15**
SP38	GB16214	serine proteases			**1.56**
SP45	GB17654	serine proteases	**-0.84**		**-1.22**
SP46	GB16367	serine proteases	**0.64**		
SP49	GB15317	serine proteases			**1.67**
SP8	GB18767	serine proteases	**-1.70**		
SPH19	GB17345	serine proteases	**-0.95**		
TEP7	GB12605	TEP			**1.21**
cact-1	GB10655	Toll/TLR			**0.41**
cact-2	GB13520	Toll/TLR			**0.81**
dorsal-1A	GB19066	Toll/TLR	**0.30**		
Dorsal-1B	GB19537	Toll/TLR			**-0.63**
Mik2	GB14720	Toll/TLR	**0.31**		
NEC LIKE	GB16472	Toll/TLR	**1.69**		**2.73**
NEC LIKE	GB17012	Toll/TLR	**-0.34**		
NEC LIKE	GB19582	Toll/TLR			**1.28**
pelle	GB16397	Toll/TLR			**0.44**
PSH LIKE / CSP14	GB14044	Toll/TLR/ serine protease	**0.67**		**1.39**
PSH LIKE / SP13	GB15640	Toll/TLR / serine protease			**0.56**
Toll (TLR)	GB18520	Toll/TLR	**0.81**		**1.03**
